# In Vitro Activity of Organoselenium Compounds Against *Trichophyton* spp.

**DOI:** 10.3390/antibiotics15101018

**Published:** 2026-10-14

**Authors:** Beatriz de Souza Cardoso, Mônica Campos dos Santos, Mariana Rodrigues Trápaga, Cecília Bittencourt Severo, Jéssica Estefania Dávila Hidalgo, Gilson Zeni, Cristina Wayne Nogueira, David A. Stevens, Melissa Orzechowski Xavier, Vanice Rodrigues Poester

**Affiliations:** 1 Programa de Pós-Graduação em Ciências das Saúde, Faculdade de Medicina (FAMED), Universidade Federal do Rio Grande (FURG), Rio Grande 96200-200, RS, Brazil; souabeatrizcardoso16@gmail.com (B.d.S.C.); monicacampos978@gmail.com (M.C.d.S.); marianartrapaga@gmail.com (M.R.T.);; 2 Hospital Universitário Dr. Miguel Riet Corrêa Jr. (HU-FURG/Empresa Brasileira de Serviços Hospitalares—EBSERH), Rio Grande 96200-190, RS, Brazil; 3 Ciências Básicas da Saúde, Parasitologia e Micologia, Universidade Federal de Ciências da Saúde de Porto Alegre (UFCSPA), Porto Alegre 90050-170, RS, Brazil; ceciliabs@ufcspa.edu.br; 4 Centro de Ciências Naturais e Exatas, Universidade Federal de Santa Maria, Santa Maria 97105-900, RS, Brazil; gzeni@ufsm.br (G.Z.); criswn@ufsm.br (C.W.N.); 5 Division of Infectious Diseases and Geographic Medicine, Stanford University School of Medicine, Stanford, CA 94305, USA; stevens@stanford.edu

**Keywords:** diphenyl diselenide, ebselen, dermatophytes, cutaneous mycoses

## Abstract

**Background/Objectives:** Dermatophytosis is among the most prevalent fungal infections worldwide, and the emergence of antifungal resistance highlights the need for novel the-rapeutic strategies. Organoselenium compounds have demonstrated promising antifungal activity against several pathogenic fungi, although their activity against dermatophytes remains poorly explored. Here we investigate the in vitro antifungal activity of four organoselenium compounds, ebselen, diphenyl diselenide [(PhSe)_2_], *p*-methoxy-diphenyl diselenide (BMD), and bis-(3-trifluoromethyl phenyl) diselenide (CF_3_). **Methods:** Eighteen clinical isolates belonging to the *T. mentagrophytes* (*n* = 10) and *T. rubrum* (*n* = 8) species complexes were studied and potential synergism with fluconazole (FCZ) also explored. Antifungal susceptibility testing and drug interaction were performed according to Clinical and Laboratory Standards Institute broth microdilution protocols and checkerboard assay, respectively. **Results:** Ebselen, (PhSe)_2_, and BMD inhibited all *Trichophyton* spp. isolates with a geometric mean (GM) of minimum inhibitory concentration (MIC) less than or equal to 6 µg/mL. CF_3_ inhibited 78% (14/18) of the isolates and had higher MICs (GM ≥ 43.5 µg/mL). Combination assays revealed synergistic interactions between FCZ and the organoselenium compounds, ranging from 45% to 89%, without antagonistic interactions. Moreover, FCZ activity was enhanced in 57–86% of resistant isolates, with BMD showing the most favorable profile in combination with FCZ. **Conclusions:** These findings demonstrate the promising antifungal potential of organoselenium compounds against the major dermatophytes associated with human infection and support further investigation of these compounds, particularly ebselen, (PhSe)_2_, and BMD, as novel antifungal agents, either alone or in combination with conventional antifungal drugs.

## 1. Introduction

Dermatophytes are keratinophilic filamentous fungi that affect approximately 25% of the global population, causing tinea corporis, tinea pedis, onychomycosis, tinea cruris, among others [1,2,3]. *Trichophyton* spp., particularly members of the *T. rubrum* and *T. mentagrophytes* species complexes, are the principal causative agents of human dermatophytosis worldwide [4,5]. Dermatophytosis generally requires prolonged treatment, frequently involving drugs with potential drug–drug interactions, which may contribute to poor treatment adherence and abandonment, particularly in onychomycosis [6,7]. In addition, the emergence of antifungal resistance among dermatophytes has become a growing public health concern [8,9,10].

These concerns highlight the need to develop new antifungal compounds against dermatophytes, particularly *Trichophyton* spp., the most common causative agents of human dermatophytosis. In this context, organoselenium compounds have emerged as promising antimicrobial and antifungal agents [11,12]. However, to the best of our knowledge, only one study has evaluated their activity against *Trichophyton* spp., reporting promising in vitro activity of ebselen and diphenyl diselenide against 37 human and animal isolates belonging to the *T. mentagrophytes* species complex [13]. In that study, ebselen exhibited the greatest activity, with a geometric mean of the minimum inhibitory concentration (MIC) of approximately 0.5 μg/mL. In contrast, diphenyl diselenide showed a geometric mean MIC of approximately 15 μg/mL. Furthermore, synergistic or additive interactions between organoselenium compounds and terbinafine or itraconazole were observed in 6–20% of the isolates. However, the interaction between organoselenium compounds and fluconazole, another antifungal agent used to treat dermatophytosis, was not evaluated [13].

Therefore, considering that structural modifications of organoselenium compounds may enhance their antifungal activity, as previously demonstrated for anti-*Sporothrix* activity using diaryl diselenide analogs [14], and given the lack of studies investigating the activity of these compounds against members of the *T. rubrum* species complex as well as their interaction with fluconazole, further investigation is warranted. In view of these gaps, the present study aimed to evaluate the in vitro activity of four organoselenium compounds against clinical isolates of *Trichophyton* spp., both alone and in combination with fluconazole.

## 2. Results

Three organoselenium compounds inhibited all *Trichophyton* spp. isolates (*n* = 18) included in the study. Ebselen and BMD caused the lowest MIC values; MIC range, 1–4 µg/mL (GM, 2.2 µg/mL; MIC_50_, 2 µg/mL; MIC_90_, 4 µg/mL); and MIC range, 2–8 µg/mL (GM, 2.6 µg/mL; MIC_50_, 2 µg/mL; MIC_90_, 4 µg/mL), respectively. The next most potent was (PhSe)_2_, MIC range, 2–16 µg/mL (GM, 6.6 µg/mL; MIC_50_, 8 µg/mL; MIC_90_, 16 µg/mL). In contrast, CF_3_ inhibited 78% (*n* = 14) of the isolates, with MIC values ranging from 8 to 64 µg/mL (GM, ≥43.5 µg/mL; MIC_50_, 64 µg/mL; MIC_90_, >64 µg/mL) (Table 1). Isolates from *T. rubrum* species complex (*n* = 8) were slightly more susceptible to the four organoselenium compounds than those from *T. mentagrophytes* species complex (*n* = 10), exhibiting lower GM MIC values, as shown in Table 1.

The fluconazole (FCZ) susceptibility of the *Trichophyton* spp. isolates (*n* = 18) showed MIC values ranging from 2 to ≥32 µg/mL, with a GM of 11.3 µg/mL. *Candida parapsilosis* ATCC CP22019 showed an MIC range of 2–4 µg/mL, in accordance with the Clinical and Laboratory Standards Institute (CLSI) M27 protocol [15]. Overall, 39% of the isolates (7/18), all belonging to the *T. mentagrophytes* species complex, were indicative of resistance to FCZ (MIC ≥ 32 µg/mL) [16]. The interactions between FCZ and the organoselenium compounds were synergistic in 89% of the combinations with BMD, 56% with CF_3_, 50% with (PhSe)_2_, and 45% with ebselen. No antagonistic interactions were observed for any combination of FCZ and the organoselenium compounds (Table 2). Among the FCZ-indicated resistant isolates, 86% (6/7) showed a reduction in MIC values for FCZ when combined with BMD, whereas 57% (4/7) showed a reduction in MIC values for FCZ when combined with ebselen, (PhSe)_2_, or CF_3_.

## 3. Discussion

Our study contributes to the search for new antifungal agents against dermatophytes by evaluating the activity of organoselenium compounds against the major species complexes associated with human dermatophytosis [2,17]. Furthermore, it expands the spectrum of activity previously reported for these compounds by including the *T. rubrum* species complex and extending the evaluation from the two organoselenium compounds previously tested against the *T. mentagrophytes* species complex [13] to four compounds. Among the tested compounds, ebselen and the structural diaryl diselenide analog, BMD, showed the lowest in vitro MIC values, consistent with the findings of Gnat et al. [13] for ebselen. Overall, three compounds, ebselen, BMD, and (PhSe)_2_, inhibited all isolates at concentrations ≤16 µg/mL (GM of <7 µg/mL).

CF_3_ was the least active compound against dermatophytes compared with the other organoselenium compounds, which have also been reported to be active against other fungal pathogens, including *Candida* spp., *Aspergillus* spp., *Fusarium* spp., and *Sporothrix* spp. [14,18]. The antifungal activity of organoselenium compounds is thought to be associated with regulation of cellular redox homeostasis [19]. Depending on their chemical structure, concentration, and molecular targets, these compounds may exhibit either antioxidant or pro-oxidant properties [20]. Therefore, structural modifications can play a key role in determining their biological activity. The introduction of a methoxy group at the para position of the aromatic ring in BMD may be associated with enhanced antifungal activity compared with the parent compound, (PhSe)_2_, in our study. In contrast, the introduction of a trifluoromethyl group at the meta position in CF_3_ apparently reduced its antifungal activity based on our data. These differences may be related to changes in physicochemical properties that influence fungal cell uptake, intracellular distribution, and target interactions. The exact mechanisms underlying these differences remain poorly understood, and further studies are required to clarify the structure–activity relationships of organoselenium compounds, as well as their pharmacokinetic and pharmacodynamic properties.

The potential of organoselenium compounds as novel antifungal agents against dermatophytes may be highlighted for both oral and topical applications. The available toxicological data support the feasibility of systemic administration. Ebselen and (PhSe)_2_ exhibit intraperitoneal LD_50_ values of 93.2 and 65.5 mg/kg, respectively, whereas BMD and CF_3_ exhibit oral LD_50_ values of >372 and 278 mg/kg, respectively, in mice [21,22,23]. In addition, administration of CF_3_ at an oral dose of 448 mg/kg resulted in the death of all animals, while (PhSe)_2_ caused the death of all animals at an intraperitoneal dose of 109.2 mg/kg. In contrast, no mortality was observed at BMD oral doses up to 372 mg/kg or at an ebselen intraperitoneal dose of 16.5 mg/kg [21,22,23]. An additional finding regarding toxicity is that CF_3_ induced DNA damage in V79 cells at doses starting from 22.4 mg/kg [24]. These values may be considered encouraging regarding the potential safety profile of these compounds, given the in vitro concentrations required to inhibit dermatophyte growth (GM ranging from 2.2 to ≥43.5 µg/mL). In addition, the acute toxicity data reported for these compounds, including the absence of mortality at doses considerably higher than those required for antifungal activity for some compounds, provide preliminary support for the feasibility of systemic administration. However, acute toxicity data alone are insufficient to establish their safety for systemic use, particularly under repeated or prolonged exposure, and further pharmacokinetic, toxicological, and efficacy studies are needed to determine their therapeutic window and adequately evaluate their safety. In addition, topical formulations could represent an attractive alternative for these cutaneous mycoses, as they may deliver higher local drug concentrations directly to the site of infection while minimizing systemic exposure and the risk of toxicity or drug interaction.

Another argument supporting the topical use of organoselenium compounds is their potential as adjuvants to commercially available antifungal drugs. In the present study, combinations of organoselenium compounds with FCZ resulted in synergism in 45% to 89% of interactions. They reversed the FCZ-resistant phenotype in 57% to 86% of the resistant isolates, with BMD showing the most promising results. Importantly, no antagonistic interactions were detected in our study. In contrast, Gnat et al. [13] reported synergistic or additive interactions between organoselenium compounds and terbinafine or itraconazole in only 6–20% of *T. mentagrophytes* isolates, whereas antagonistic interactions were observed in 8–81% of the isolates, depending on the compound–drug combination. FICi cutoff values are not standardized in the literature or across protocols [13,14,25,26,27,28,29,30,31,32,33,34].

These findings indicate that no antagonistic interactions were observed between the organoselenium compounds and FCZ under the conditions evaluated in our study. However, the potential interactions of these compounds with itraconazole and terbinafine warrant further investigation, particularly against isolates belonging to the *T. rubrum* species complex, which were not included in the previous study evaluating these drug combinations [13]. Combining organoselenium compounds with commercially available antifungal drugs may represent an attractive therapeutic strategy, particularly through topical administration of organoselenium compounds associated with systemic treatment using conventional antifungal agents. Such an approach could enhance therapeutic efficacy while minimizing systemic exposure to organoselenium compounds, particularly by reducing treatment duration and, consequently, treatment abandonment, especially in cases of onychomycosis, the clinical manifestation that typically requires the longest treatment duration [2,3]. However, further pharmacological, preclinical, and clinical studies are required to confirm the efficacy, safety, and therapeutic potential of these combinations, particularly in topical formulations for the treatment of dermatophytosis.

Some limitations of our study should be acknowledged. First, the number of isolates tested, which was less than 20 and recovered from a single hospital/region, limits the ability to extrapolate the results for all of the clinical isolates of *T. rubrum* and *T. mentagrophytes* complex strains. In addition, the combination assays were performed exclusively with FCZ, limiting the extrapolation of the results to other clinically relevant antifungal agents. Although previous studies have evaluated combinations of organoselenium compounds with terbinafine and itraconazole, additional investigations, including different dermatophyte species, are still needed. Future studies should evaluate the effects of these compounds on dermatophyte cells, including membrane integrity, oxidative stress, mitochondrial function, biofilm formation, and other cellular pathways, and validate their efficacy and safety in appropriate preclinical models. In addition, future studies should evaluate the activity of organoselenium compounds against the emerging sexually transmitted *Trichophyton* dermatophytoses, including *T. mentagrophytes* ITS genotype VII and *T. indotineae*, which represent emerging challenges in dermatophyte epidemiology and antifungal resistance [35].

## 4. Materials and Methods

**Figure 1:**
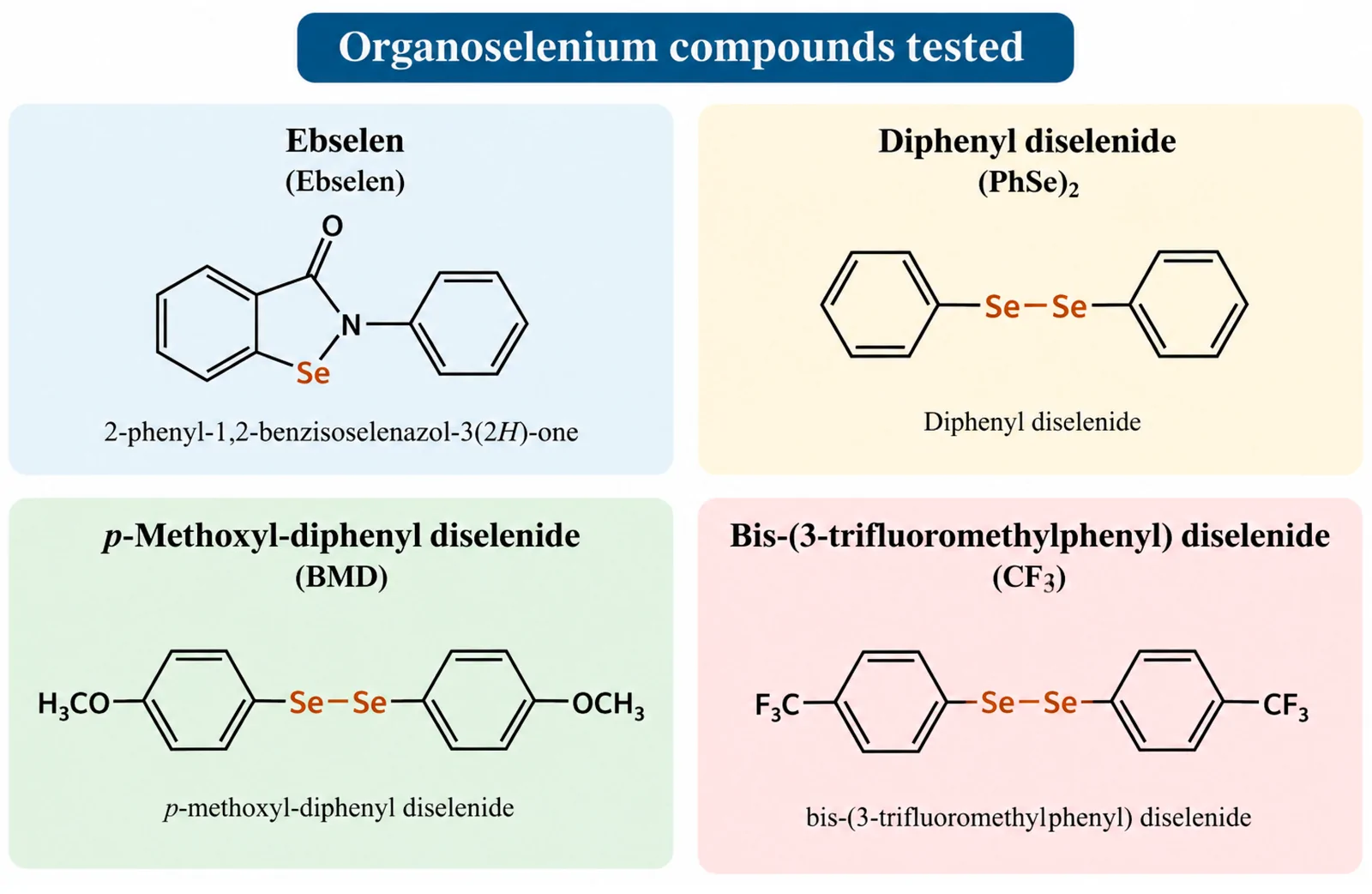
Chemical structures of the organoselenium compounds tested in vitro against *T. mentagrophytes* species complex (*n* = 10) and *T. rubrum* species complex (*n* = 8): ebselen, diphenyl diselenide (PhSe)_2_, *p*-methoxyl-diphenyl diselenide (BMD), and bis-(3-trifluoromethylphenyl) diselenide (CF_3_). Figure created using ChatGPT (GPT-6, OpenAI).

A total of 18 clinical human isolates of *Trichophyton* spp., including members of the *T. mentagrophytes* species complex (*n* = 10) and *T. rubrum* species complex (*n* = 8) was used to evaluate the in vitro inhibitory activity of ebselen, diphenyl diselenide (PhSe)_2_, and two diaryl diselenide analogs, *p*-methoxyl-diphenyl diselenide (BMD) and bis-(3-trifluoromethylphenyl) diselenide (CF_3_) (Figure 1). Isolates were previously identified based on protein profiles using matrix-assisted laser desorption/ionization time-of-flight mass spectrometry (MALDI-TOF MS; Bruker^®^, Billerica, MA, USA).

FCZ (Sanobiol^®^, Pouso Alegre, Minas Gerais, Brazil) was obtained commercially. Organoselenium compounds were synthesized through a collaboration with the Department of Biochemistry and Molecular Biology at the Federal University of Santa Maria. BMD and CF_3_ were synthesized using the Grignard reaction, yielding final HPLC purities of 99% and 98%, respectively. (PhSe)_2_ was synthesized according to the method described by Paulmier [36], whereas ebselen was synthesized following the procedure reported by Engman and Hallberg [37]. Drugs were tested at final concentrations ranging from 0.125 to 32 μg/mL for FCZ, 0.5 to 32 μg/mL for ebselen and BMD, and 1 to 64 μg/mL for (PhSe)_2_ and CF_3_. Organoselenium compounds were prepared as 100× concentrated stock solutions in dimethyl sulfoxide, whereas FCZ was directly diluted in RPMI 1640 medium.

The in vitro activity of the compounds against *Trichophyton* spp. was evaluated according to the CLSI M38 protocol [38]. Briefly, *Trichophyton* spp. strains were cultured on potato dextrose agar tubes at 25 °C for seven days. Conidia were harvested and suspended in sterile distilled water, and the inoculum concentration was adjusted to (1 to 3) × 10^5^ conidia/mL using a Neubauer chamber. The suspension was then diluted 1:100 in RPMI 1640 broth to obtain a final inoculum concentration of (1 to 3) × 10^3^ conidia/mL [38]. Drugs were diluted in RPMI 1640 medium at a 1:50 ratio and dispensed into microplates, along with the standardized inocula to achieve the final concentrations tested. The pour-plate technique confirmed the inoculum concentration. In addition, a drug quality control was included using FCZ against *Candida parapsilosis* ATCC CP22019 [15]. The microplates were incubated at 35 °C for 96 h. After incubation, the MIC of the organoselenium compounds was defined as the lowest concentration that completely (100%) inhibited fungal growth, whereas the MIC of FCZ was defined as the concentration producing 80% inhibition of fungal growth in the individual activity, in accordance with the CLSI M38 guidelines [38]. For the combination assays, the endpoint was defined as 100% inhibition of fungal growth. For the evaluation of the activity of compounds alone, the geometric mean (GM) MIC, MIC_50_, and MIC_90_ values, corresponding to the concentrations required to inhibit 50% and 90% of the isolates, respectively, were calculated for each compound. For isolates in which a compound did not achieve 100% inhibition at the highest concentration tested, the MIC was extrapolated to the next higher concentration to calculate the geometric mean.

The interaction between FCZ and organoselenium compounds was evaluated using the checkerboard assay [13,39,40]. Inoculum preparation, drug concentration ranges, and incubation conditions were performed as described above. Drug interactions were assessed by calculating the fractional inhibitory concentration index (FICi). Interactions were classified as synergism, subclassified into strong synergism (SS; FICi < 0.5), weak synergism (WS; 0.5 ≤ FICi < 1); indifferent (IND; 1 ≤ FICi ≤ 2); or antagonistic (ANT; FICi > 2), as previously detailed [14,25,26,27,28,29,30,31,32,33,34]. In addition to evaluating FCZ activity, the GM of MICs and the percentage of isolates with MIC values ≥32 µg/mL, suggestive of FCZ resistance, were calculated [16].

## 5. Conclusions

In conclusion, our study provides important pre-clinical evidence regarding the in vitro antifungal activity of organoselenium compounds against the two major dermatophyte species complexes associated with human dermatophytosis. Among the evaluated compounds, ebselen, a repurposed drug with an established safety profile, and BMD exhibited the greatest in vitro activity and may represent the most promising candidates for further translational studies. Among the combinations evaluated, BMD associated with FCZ showed the most promising activity, supporting further investigation in in vivo models using this compound alone and in association with FCZ.

## Figures and Tables

**Table 1:** In vitro activity of four organoselenium compounds, ebselen, diphenyl diselenide (PhSe)_2_, *p*-methoxyl-diphenyl diselenide (BMD), and bis-(3-trifluoromethylphenyl) diselenide (CF_3_), against the *Trichophyton mentagrophytes* species complex (*n* = 10) and the *T. rubrum* species complex (*n* = 8). Results are expressed as minimum inhibitory concentration (MIC) ranges and geometric mean MIC (GM MIC) values (µg/mL).

Compound	*T. mentagrophytes* MIC Range (GM, µg/mL)	*T. rubrum* MIC Range (GM, µg/mL)	Overall MIC Range (GM, µg/mL)
Ebselen	1–4 (2.5)	1–4 (1.8)	1–4 (2.2)
(PhSe)_2_	4–16 (8)	2–8 (5.2)	2–16 (6.6)
BMD	2–8 (3.2)	2 (2.0)	2–8 (2.6)
CF_3_	8–≥64 (≥52.0)	8–≥64 (≥34.9)	8–≥64 (≥43.5)

**Table 2:** In vitro interactions between fluconazole (FCZ) and four organoselenium compounds, ebselen, diphenyl diselenide (PhSe)_2_, *p*-methoxyl-diphenyl diselenide (BMD), and bis-(3-trifluoromethylphenyl) diselenide (CF_3_), against *Trichophyton* spp. (*n* = 18), expressed as fractional inhibitory concentration index (FICi) values and interaction profiles.

Combination *	SS % (*n)*	WS % (*n)*	IND % (*n)*	ANT % (*n)*
Eb + FCZ	6 (1)	39 (7)	55 (10)	0 (0)
(PhSe)_2_ + FCZ	0 (0)	50 (9)	50 (9)	0 (0)
BMD + FCZ	0 (0)	89 (16)	11 (2)	0 (0)
CF_3_ + FCZ	6 (1)	50 (9)	44 (8)	0 (0)

* Interpretation of fractional inhibitory concentration index (FICi) values: strong synergism (SS; FICi < 0.5), weak synergism (WS; 0.5 ≤ FICi < 1), indifferent (IND; 1 ≤ FICi ≤ 2), or antagonistic (ANT; FICi > 2).

## Data Availability

No additional data were generated in this study; all data are available in the article.
